# Detectability of *Plasmodium falciparum *clones

**DOI:** 10.1186/1475-2875-9-234

**Published:** 2010-08-18

**Authors:** Michael T Bretscher, Francesca Valsangiacomo, Seth Owusu-Agyei, Melissa A Penny, Ingrid Felger, Tom Smith

**Affiliations:** 1 Swiss TPH, Basel, Switzerland; 2 University of Basel, Basel, Switzerland; 3 Kintampo Health Research Centre, Kintampo, Ghana

## Abstract

**Background:**

In areas of high transmission people often harbour multiple clones of *Plasmodium falciparum*, but even PCR-based diagnostic methods can only detect a fraction (the detectability, *q*) of all clones present in a host. Accurate measurements of detectability are desirable since it affects estimates of multiplicity of infection, prevalence, and frequency of breakthrough infections in clinical drug trials. Detectability can be estimated by typing repeated samples from the same host but it has been unclear what should be the time interval between the samples and how the data should be analysed.

**Methods:**

A longitudinal molecular study was conducted in the Kassena-Nankana district in northern Ghana. From each of the 80 participants, four finger prick samples were collected over a period of 8 days, and tested for presence of different Merozoite Surface Protein (msp) 2 genotypes. Implications for estimating *q *were derived from these data by comparing the fit of statistical models of serial dependence and over-dispersion.

**Results:**

The distribution of the frequencies of detection for msp2 genotypes was close to binomial if the time span between consecutive blood samples was at least 7 days. For shorter intervals the probabilities of detection were positively correlated, i.e. the shorter the interval between two blood collections, the more likely the diagnostic results matched for a particular genotype. Estimates of *q *were rather insensitive to the statistical model fitted.

**Conclusions:**

A simple algorithm based on analysing blood samples collected 7 days apart is justified for generating robust estimates of detectability. The finding of positive correlation of detection probabilities for short time intervals argues against imperfect detection being directly linked to the 48-hour periodicity of *P*. *falciparum*. The results suggest that the detectability of a given parasite clone changes over time, at an unknown rate, but fast enough to regard blood samples taken one week apart as statistically independent.

## Background

In areas of high endemicity of *Plasmodium falciparum*, human hosts are often superinfected with multiple clones of the parasite [1]. Identification of these concurrent infections is important for understanding patterns of drug resistance [2] and of the transmission of the parasite. PCR-based methods for detecting parasites not only have lower detection limits than blood smear microscopy, but also make it possible to distinguish genetically distinct clones, and hence to compute multiplicity of infection. But at least two diagnostic problems remain: i) the same host might be infected with more than one parasite clone of the same genotype, which can introduce bias into estimates of multiplicity of infection [3]. ii) PCR detection can be negative because the sample taken does not contain any parasites. This may happen due to effects of acquired immunity or synchronization of the parasite population. Failure to account for imperfect detection biases several standard epidemiological measures, such as prevalence and multiplicity of infection. Most critically, analysis of drug failure rates using molecular typing may overlook breakthrough parasite clones or conversely misclassify them as new infections after treatment. Repeated blood samples from the same host can be analysed to estimate the probability that a clone is detected in any given sample (the detectability, *q*). For microscopy data, where individual parasite clones cannot be distinguished, the statistical methods of [4,5] are applicable. Both assume infections are neither acquired nor cleared during the study. For molecular data, several pieces of work aiming at estimating infection duration and force of infection also yielded measurements of detectability and its dependence on age of the host [1,6-8]. These methods make use of data collected over longer time periods (several months up to a year), with surveys every 1 to 2 months, the kind of which may not be easily available in practice. Moreover, the obtained estimates of detectability depend on simultaneous estimates of infection and recovery rates as well as on assumptions concerning these processes. A simple method is therefore presented, to estimate the detectability of infecting clones from molecular data with short inter-survey intervals. It makes use of pairs of surveys sufficiently close in time, such that reinfection with the same parasite genotype can be safely excluded. The method is similar to the one presented in [4], but adapted for the context of molecular diagnostic methods. This implies that the maximal number of "infections" is not limited by the number of hosts in the study, but rather represents individual parasite clones. The methods of [1,6-8] as well as the one presented here assume that the detections of an infecting clone at different time points are independent from each other. While it seems reasonable to make such an assumption, provided intervals between surveys are long enough, it is not clear how long these intervals need to be. Numerous publications report complicated periodic behaviour of fevers or parasitaemia [9], or detection events [10], which creates a need to establish the circumstances under which the methods mentioned above can be applied.

In order to evaluate the effect of possible "nonrandom" behaviour of clonal infections on estimates of detectability, a longitudinal study comprising 80 individuals was conducted in northern Ghana. From each participant, four blood samples were collected over a period of 8 days. Using these data, various statistical models are compared with respect to their goodness of fit, and a series of hypothesis tests is performed. The resulting statistical description of the within-host dynamics of *P*. *falciparum *clones, as observed by molecular typing methods, allows us to justify a simple algorithm for obtaining reasonably robust estimates of *q *and specify the circumstances under which this method is applicable.

## Methods

### Study site and sample collection

**Figure 1:**
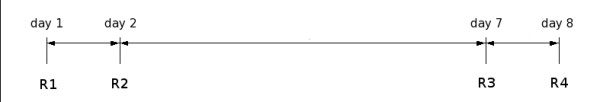
**Study design**. Blood samples were collected in four survey rounds (R1-R4), on day 1, 2, 7 and 8. The result of this study design are two sampling intervals of 1 day, one of 5, two of 6, and one of 7 days. A 48-hour periodicity of *P*. *falciparum *detectability could therefore be identified, as it should show positive correlation of detection outcomes between surveys with even-numbered interval length, and negative correlation between surveys with odd-numbered interval length (in days).

The present survey was conducted following a one year longitudinal study on malaria epidemiology [7,8,11,12] in the Kassena-Nankana district (KND), in the Upper East Region of Ghana. The malariological situation in this area is characterized by very high prevalence and multiplicity of infection [11,13], and year-round transmission with seasonal variation in transmission intensity [8]. From the participants of the mentioned main study, 80 individuals below 20 years of age were randomly selected for this followup. From these, a total of four blood samples were taken on the last survey of the main study as well as 1, 6 and 7 days later (Figure 1). The present analysis was restricted to these four samples within eight days. Study participants were visited in the early mornings of each day and houses were visited in the approximately the same order, to ensure sample collection at roughly the same time of day for each individual. Whole blood was collected on "ISOCode™Stix" PCR template preparation dipsticks (Schleicher & Schuell, Dassel, Germany). Study participants who were sick at the time of the survey were referred to the routine health services. No anti-malarial treatments were administered by the research team.

### Genotyping

DNA was eluted from "ISOCode™Stix" filter paper and screened for presence of *P*. *falciparum *by polymerase chain reaction (PCR). Sample processing and PCR conditions have been described in detail [14]. In brief, all samples were subjected to PCR using primers specific for the merozoite surface protein (msp) 2 locus. Genotypes were distinguished on the basis of length polymorphism and PCR fragments were precisely sized by automated capillary electrophoresis and GeneMapper^® ^software. An inhouse generated software identified all genotypes per sample and transformed the data into different formats suitable for data management and statistical analysis. Given the high number of msp2 genotypes in the population, re-infection with the same genotype was assumed to be a rare event. As a consequence of this, for any given host, msp2 genotype is assumed to be synonymous with "infecting clone" in all analyses.

### Data analysis

Only data of those participants who were present at all four survey rounds, and where at least one genotype was found, were included in the analysis. This reduced the number of individuals in the data set to 69. Patterns of appearance and disappearance of specific parasite genotypes depend on rates of infection and clearance as well as on detectability. However, for the purpose of the present analysis, acquisition and loss of infections were neglected. It was assumed that there are no false positive results and that an infecting clone is present throughout all four surveys if detected at least once. This is justified by the comparatively short time interval between the first and the last survey, and by previously published estimates of infection and clearance rates from the dataset of the main study [8]:

According to the authors, a person experienced an estimated 0.6 new infections during the time of the study (31 new infections per annum in the corresponding season). This implies that around 0.6 _* _69 ≈ 41 or approximately 8% of the 519 clones in the data set may have been acquired during the study. Similarly, assuming an average (clonal) infection duration of 150 days and that infections were acquired at random times relative to the time of the study leads to an estimate of 7/150 ≈ 5% of clones being cleared during the study period of seven days. Failure or success to detect a strain was denoted by 0 or 1, respectively, yielding 519 binary sequences of length four. The 15 possible sequence types containing at least one positive test result are referred to by the binary number they encode (Table 1). The resulting pool of sequences was either analysed as a whole, or split into the following age-groups (age in years): 0-2, 3-5, 6-10, 11-15, 16-20. This mode of analysis implies that clones infecting the same host are assumed independent of each other. Further, the present analysis is only concerned with variation in detectability among clones, not among hosts.

**Table 1:** Data coding

R1	R2	R3	R4	**sequence no**.	count
0	0	0	0	0	-
0	0	0	1	1	43
0	0	1	0	2	42
0	0	1	1	3	26
0	1	0	0	4	54
0	1	0	1	5	10
0	1	1	0	6	13
0	1	1	1	7	19
1	0	0	0	8	28
1	0	0	1	9	21
1	0	1	0	10	41
1	0	1	1	11	34
1	1	0	0	12	64
1	1	0	1	13	22
1	1	1	0	14	41
1	1	1	1	15	61

Failure or success to detect a clone at any given survey round was coded using binary notation. This yielded 519 sequences of length four. Sequences were numbered according to the binary value they encode. Sequence no. 0 is invisible.

A series of *χ*^2 ^tests and Spearman's rank correlation analysis yielded qualitative information on thetemporal behavior of detectability. Further, a series of models for the dynamics of detectability were fit to the data using Bayesian MCMC. These are de-scribed in detail below. The models and their estimates of detectability are compared using Deviance Information Criterion (DIC) as measure of goodness of fit [15]. That only sequences with at least one positive result were included in the data, and therefore the data are biased, was accounted for in all analyses. The software Winbugs [16] was used for all Bayesian model fitting, whereas for all other analyses the software package R was used [17].

### Models of detection

In order to explore the short term dynamics of detectability, three statistical models are compared with regard to their goodness of fit (M1 to M3 be low). These models are in the form of an expression for the detectability of clone *i *at time point *t*. This allows for fitting of the models by Bayesian Markov Chain Monte Carlo (MCMC), assuming individual detections are Bernoulli-distributed as

$$X_{i,t}\sim Bern(q_{i,t}^{\text{o}bs}).$$

In addition, a simple method of directly measuring detectability from pairs of surveys (M0) is used. Applying this method to all available survey pairs in the data set and comparing the estimates of *q *with the model results allows us to develop criteria for the circumstances under which the method may be used.

#### M0: Direct estimation of detectability

Following [4], a method is proposed for direct estimation of the detectability *q *from pairs of observations. The estimate is a function of the number of infecting clones that were detected in only one of two survey rounds (*n*_1_), and the number which was detected in both (*n*_2_). Assuming a binomial distribution of the number of times a clone is detected and correcting for the detection bias leads to the following expression for the estimated detectability $\overset{\wedge }{q}$ so given by

(1) $$\overset{\wedge }{q}=\frac{2n_{2}}{n_{1}+2n_{2}}.$$

The binomial likelihood model implies statistical independence of detections at different time points. This assumption might be violated if detectability exhibits temporally structured behavior. The detectability model underlying this method is identical to M1 below, but this method only uses two observations, and model fitting in order to estimate *q *is done analytically.

This simple method is compared to models M1 to M3 (below) in order to justify it's use, and to establish the conditions where it can be applied. For a formal derivation of (1) and confidence intervals for $\overset{\wedge }{q}$ please refer to the appendix (additional file 1).

#### M1: Binomial model

Model 1 follows M0 in assuming that the detectability *q_i,t _*is a constant for all clones *i *and time points *t*, namely

$$q_{i,t}=\bar{q}.$$

This implies independence of detecting a clone at time *t *from whether it was detected at other time points, and homogeneity of the infection population with respect to detectability.

#### M2: Beta-binomial model

Model 2 allows for variation in detectability among clones, but requires every clone to have the same detectability throughout the study. Variation in detectability is modeled using a beta distribution:

$$q_{i,t}=q_{i}\sim Beta(a,b),$$

where *a *and *b *are the shape parameters of the beta distribution.

#### M3: First order Markov Chain

Model 3 uses a two-state, first order Markov chain to represent the time evolution of detectability. In a first order Markov chain, the probability of detecting a clone at time *t *depends on whether it was detected at time *t*-1. This is achieved by defining *q_i,t _*as one of two detectabilities *q*_1 _or *q*_0_, depending on whether the clone *i *was detected at time *t *- 1, or not.

$$q_{i,t}=\{\begin{array}{ll} q_{1} & ,\text{if}\;\text{clone}\;\;\text{detected}\;\text{at}\;t-1\\ q_{0} & ,\text{otherwise} \end{array}$$

This is equivalent to a two-state Markov chain defined by the transition matrix

$$T=\left(\begin{array}{cc} t_{00} & t_{01}\\ t_{10} & t_{11} \end{array}\right)\;=\left(\begin{array}{ccc} 1- & q_{0} & q_{0}\\ 1- & q_{1} & q_{1} \end{array}\right),$$

where *t_i,j _*is the probability that a transition from state *i *to state *j *occurs in the data, when the two observations are one time unit apart (24 h in this case). If the two observations are *n *days apart, the the transition matrix is raised to the power of *n *and becomes

$$T_{n}=T^{n}.$$

The probability of detecting a clone at the first survey, *q*_*i*,1_, was assumed to be equal to the expected detectability $\bar{q}$, which follows from the stationary distribution of the Markov chain defined by *T*. In equilibrium, the the number of transitions from 0 to 1 and from 1 to 0, respectively, must be equal. Therefore $\bar{q}t_{10}=(1-\bar{q})t_{01}$, which leads to the expression for $\bar{q}$ as given by

$$\bar{q}=\frac{t_{01}}{t_{01}+t_{10}}=\frac{q_{0}}{1+q_{0}-q_{1}}.$$

An important feature of this simple model is that it not only represents a random walk in "detection space" (i.e. switching between being detected and not being detected), but that it can also be interpreted as a random walk in "detectability space" (switching between the two detectabilites). This can be illustrated as follows: The probability that a clone changes its internal state from *q*_0 _to *q*_1 _is equal to the probability that it is detected while being in state *q*_0_, which is equal to *q*_0_. Likewise, the probability of a transition from *q*_1 _to *q*_0 _is 1 - *q*_1_. The resulting transition matrix for such a process is identical to T. However, since this model is fitted to a population of sequences, as opposed to just fitting it to one single time-series, one has to be careful in interpreting a possible best fit of this model: simple heterogeneity in detectability among clonal infections would also result in different estimates for *q*_1 _and *q*_0_, even if there is no random walk in detectability within a single clone. Therefore, only in combination with the results of M2, which is also able to capture such heterogeneity, is one able to interpret *t*_10 _and *t*_01 _as transition probabilities between *q*_0 _and *q*_1_.

### Bias correction of detectability estimates

An "observed" detectability estimated by fitting these models does not correspond to the underlying "true" detectability because clones only appear in the data if detected at least once. Therefore, a bias correction is required in order to estimate the true detectability *q^true^*. This was achieved as follows: by considering only the (time independent) mean detectability $q^{obs}=E[q_{i,t}^{obs}]$, the corresponding mean true detectability *q^true ^*is approximated by *q^obs ^*times the probability that a clone is included in the data, so given by

$$q^{true}\approx q^{obs}(1-{\left(1-q^{true}\right)}^{4}).$$

This expression can be solved numerically by starting with the approximation

$$q^{true}\approx q^{obs}(1-{\left(1-q^{obs}\right)}^{4}),$$

and iteratively approaching *q^true^*. The magnitude of the bias in detectability estimates can thus be examined numerically. It amounts to approximately 10^-2 ^for the values of *q *in the present data. The approach was used in all models to correct the measured detectabilities for detection bias. Similarly, the true number of clones present, *N_true_*, is approximated as

$$N_{true}\approx \frac{N_{obs}}{\left(1-{\left(1-q^{true}\right)}^{4}\right)},$$

with *N_obs _*= 519.

## Results

In the complete study population (80 individuals), the average prevalence across all four survey rounds was 46% by microscopy and 0.69% by PCR. The dataset used for statistical analyses comprised 69 parasite-positive individuals between 6 months and 20 years of age, with a median age of 5.2 years (interquartile range 3.5-9.7). The median multiplicity of infection (MOI) among these was 10 (inter-quartile range 7-13), when pooling all four observations from each individual. This differs from standard practice when reporting MOI, but was justified given the very short interval between the surveys. The obtained value is expected to be a better estimate of the true MOI. When only considering single survey rounds, the median MOI among PCR-positives was 4. The 519 detected clones belonged to 77 different msp2 genotypes, with the most common allele reaching a frequency of 9.2%.

### Tests of proportion and correlation

A series of hypothesis tests was performed in order to gain insight into the statistical properties of the data-generating process. These do not relate to the models M1-M3 directly, but rather aim to look at similar questions using a completely different methodology. Any conclusions would need to be consistent with both approaches. In this analysis, the detection bias is accounted for by adding a total of 26 all-zero sequences to the data set, such that the total number of sequences equals 545. This is the "true" number of clonal infections, as estimated robustly by models M1 to M3. In the following list, *H*_1_-*H*_5 _indicate the hypotheses tested, and the corresponding p-values obtained using *χ*^2 ^tests (with the exception of the Spearman's Rank Correlation analysis) are given:

*H*_1_: All 4 surveys have an equal proportion of positive results, i.e. $\sum d_{i,1}=\sum d_{i,2}=\sum d_{i,3}=\sum d_{i,4}$ (Data: 312, 284, 277, 236). This hypothesis of stationarity is rejected by a *χ*^2 ^test with 3 degrees of freedom: P-value < 0.0001.

*H*_2_: The frequencies *s_i _*of the 16 binary sequences (including the added all-zero sequence) are multinomially distributed with expectations $s_{i}=545q^{o_{i}}{\left(1-q\right)}^{4-0_{i}}$, where *o_i _*is the number of positive testing results in sequence *i*). This hypothesis, effectively proposing that a Bernoulli-process is able to perfectly describe the data, is rejected by a *χ*^2 ^test with 14 degrees of freedom: P-Value < 0.0001.

*H*_3_: The number of sequences with *i *= 0, 1, .., 4 detections are multinomially distributed with expectations $s_{i}=545{(}_{0_{i}}^{4})q^{o_{i}}{\left(1-q\right)}^{4-0_{i}}.$ This is a slightly relaxed version of *H*_2_, such that the time order of detections is neglected, and sequences with a certain number of detections are pooled. However, this hypothesis is rejected by a *χ*^2 ^test with 3 degrees of freedom: P-Value < 0.0001.

*H*_4_: The frequencies of all four possible results of a survey pair (i.e. "00","01","10" and "11") are multinomially distributed with expectations $s_{i}=545q^{o_{i}}{\left(1-q\right)}^{2-0_{i}}.$ This is a special case of *H*_2_, only applied to a pair of surveys, instead of the whole dataset. Except for survey pair 2-3 (p-value 0.06) this hypothesis is rejected on all survey pairs by *χ*^2 ^tests with 2 degrees of freedom: P-values < 0.0001.

*H*_5_: The distribution of the number of successful detections in pairs of surveys is binomially distributed. This is very similar to *H*_3_, except that only pairs of surveys are considered. The duration between the observations turns out to be important, as the hypothesis is rejected by *χ*^2 ^tests with 1 degree of freedom on all survey pairs (p-value < 0.0001), except on the longest interval with a duration of 7 days (p-value: 0.83). The p-values of all pairs are listed in Table 2. This result is particularly interesting as it could be interpreted as test of an ergodic hypothesis, which implies that after enough time has passed, the system "forgets" where it started and its state at the second observation is independent from the first observation.

**Table 2:** Direct estimation of *q *on all survey pairs, using M0

survey pair	interval (days)	*n*_1_	*n*_2_	$\overset{\wedge }{q}$	95% CI	p-value
1-2	1	220	188	0.63	0.59-0.68	9.4e-12
3-4	1	233	140	0.55	0.49-0.60	9.1e-7
2-3	4	293	134	0.48	0.43-0.53	1.1e-2
1-3	5	235	177	0.60	0.55-0.65	2.4e-7
2-4	5	276	112	0.45	0.39-0.50	1.7e-3
**1-4**	**7**	**272**	**138**	**0.50**	**0.45-0.55**	**0.83**

Direct estimation of *q *on all pairs of surveys gave heterogeneous results. However, only in pair 1-4 are the proportions of single and double positives compatible with the binomial assumption of the direct estimation method (p-value:0.83). The estimate of *q *from this pair is close to the estimate of M3.

A Spearman's Rank Correlation analysis showed significant positive correlation between all pairs of surveys which are 24 h apart, and no correlation for all other pairs, with the exception of the pair formed of surveys 1 and 3.

### Model comparison

**Figure 2:**
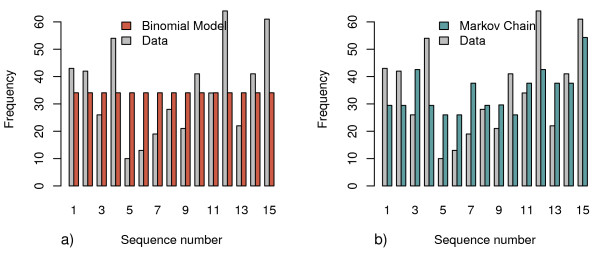
**Expected and actual frequencies of sequence types**. Comparison of sequence type frequencies in the data with their expectations from a) the binomial model (M1), and b) the Markov Chain model (M3). M3 fitted the data better, yet did not fully explain it. The beta-binomial model (M2) is not shown since it measured almost no variation in detectability among clones, and therefore effectively reduced to M1.

A comparison of models M1 to M3 with respect to their goodness of fit as indicated by lower values of DIC reveals that M3 fits the data best (Table 3). This was the case both when splitting the data by age group (DIC 2860.0) and without doing so (DIC 2856.4). The model estimating a separate parameter set for each age group indicates a decreasing trend in detectability with age, as observed by others [1,8,18]. However, the model with only one parameter set for all ages has a lower value of DIC, and therefore this trend is not significant. There is almost no difference in goodness of fit between the binomial (M1) and beta binomial models (M2), as indicated by the corresponding DIC values (all between 2870.2 and 2872.3). This is in line with the finding that the beta-binomial models estimated almost no variation in *q *and therefore effectively reduced to the corresponding binomial model. A graphical comparison of M1 and M3 is presented in Figure 2.

**Table 3:** Comparison of Models M1-M3

model	age groups	$\bar{q}$			DIC
			
M1 (binomial)	1	0.50			2870.2
M1 (binomial)	5	0.55, 0.52, 0.49, 0.53, 0.41			2872.3
		
model	Age groups	$\bar{q}$	var(*q*)		DIC
		
M2 (beta-bin.)	1	0.51	0.003		2871.5
M2 (beta-bin.)	5	0.51, 0.46, 0.46, 0.47, 0.46	0.004,0.005,0.005,0.005,0.005		2871.3
Model	age groups	$\bar{q}$	*q*_0_	*q*_1_	DIC

M3 (Markov)	1	0.50	0.47	0.59	2856.4
M3 (Markov)	5	0.55, 0.53,0.49, 0.53, 0.41	0.45, 0.44, 0.47, 0.56, 0.46	0.65, 0.64, 0.56, 0.55, 0.45	2860.0

Model results: M3 without age groups fitted the data best, indicated by it's lowest value of DIC. M2 effectively reduced to M1, as it estimated very low variance of detectability. All models estimated a true number of clones of approx. 546 (not shown), and similar values for the mean detectability $\bar{q}$ Values of $\bar{q}$ represent the bias-corrected mean detectability, whereas *q*_0 _and *q*_1 _are not bias corrected, but represent "observed" detectabilities (see section "bias correction").

### Estimates of q

The estimates of detectability (Table 3) were found to be similar for models M1 through M3, especially when common parameters for all age groups were estimated. All values were approximately 0.5, and showed little prior sensitivity. Although measuring separate detectabilities for every age group did not improve model fit, a decreasing trend of detectability with age was observed. Estimates of *q *for the youngest group are between 0.51 and 0.55, and decrease to values between 0.41 an 0.46 for the oldest age group. This is consistent with the findings of other authors [8]. Estimates of *q *obtained using M0, however, show some variation, with values ranging from 0.45 to 0.63. Table 2 shows the corresponding estimates obtained from all available pairs of surveys. Only for the measurement using survey pair 1-4 were the criteria for using the method fulfilled, as the corresponding p-value of 0.83 indicates, and the value of *q *estimated from this pair matches the estimates from the models very well.

## Discussion

The short term dynamics of asymptomatic *P*. *falciparum *clonal infections *in vivo *were characterized in order to find a simple way of measuring detectability in the field. A series of statistical tests as well as a progression through three simple models provided insight into some statistical properties of within-host dynamics monitored by molecular typing. Classical PCR ignores absolute parasite densities, but length polymorphic amplicons make it possible to distinguish between co-infecting parasite clones. Detectability, however, can be used as a proxy for parasite densities, as the two must be correlated. Since key epidemiological measures such as prevalence and multiplicity of infection (MOI) depend on the numerical value of detectability, planning and monitoring of malaria interventions rely on accurate measurements of detectability. It is important to note that detectability may not only depend on host or parasite factors, but also on the methods for generation of genotyping data. These include the method for collecting blood samples, the actual volumes of blood collected, storage conditions, PCR conditions, and competition within the PCR assay limiting the detection of minority clones. The present analysis did not consider any of these factors, but rather assumed that their impact is more or less identical for all samples and clones.

### Within-host dynamics

Measurement of detectability from longitudinal data will for practical reasons rely on binomial models of detection. This creates a need to establish under what conditions such binomial models are applicable. Different hypotheses tested on the typing data showed complicated dynamics of clonal infections for short timescales. These dynamics could not be described by a binomial model (rejection of hypotheses *H*_1 _to *H*_3_). A hypothesis which could not always be rejected was *H*_5_. This hypothesis stated that the number of successful detections in sample pairs were binomially distributed. It was not rejected for the sample pair collected at the most distant dates, i.e. from surveys 1 and 4 with interval of 7 days (Table 2). This finding could indicate that the processes governing detectability on short time scales are prone to stochastic variation such that the effect of the initial state of a clone vanishes after some time, and the two observations become independent. In other words, the corresponding test could be interpreted as test of an ergodic hypothesis, which implies that the system under investigation "forgets" it's initial state after enough time has passed. That the frequencies of "01" and "10" sequences are not equal, and therefore *H*_4 _(stricter than *H*_5_) is rejected on all survey pairs except pair 2-3, questions this interpretation, and can not be explained it in a satisfactory way. Since there are consistently more "10" pairs than '01", one could presume that the detectability of clones simply decreases with time, which would also explain the decreasing trend in the number of detections per survey (*H*_1_). However, this is mere speculation and can hardly be shown from this dataset. Nevertheless, the method of directly measuring detectability is presumably little affected by this phenomenon, as it's estimates of q only depend on the sum of single positive pairs and the obtained numerical values of *q *agree very well with the results of the other models.

Intuitively, one would expect a certain amount of variation in detectability among clonal infections, especially since these were pooled across individuals. It is therefore surprising that M2, which would allow for such variation, measured zero variance of *q *and effectively reduced to the binomial model M1. The best fitting model M3 offers a possible interpretation, as it is capable of capturing change in detectability over time. M3 models the time evolution of detectability as a Markov chain, which is equivalent to assume that a clone has detectability *q*_0 _if it was not detected on the preceding survey, and detectability *q*_1 _if it was. The obtained estimates of *q*_0 _and *q*_1 _as 0.47 and 0.59, respectively, could either indicate variation in the dataset with respect to detectability or that the detectability of a clone performs a random walk in detectability space, alternating between the two states *q*_0 _and *q*_1_. Since M2 reduced to M1, and estimated practically no variation in detectability, we have to assume the latter.

One might expect parasite densities to fluctuate with a period of approximately 48 hours, as observed in malariatherapy-data [9], and in good agreement with in-vitro measurements of a 48 hour erythrocytic cycle. In fact, such periodic behaviour of asymptomatic infections has been reported [10,19]. The present analysis does not find a 48-hour periodicity, rather the opposite: both the best fitting model as well as the results of the Spearman's rank correlation analysis indicate positive autocorrelation between time points which are 24 hours apart. A process with a periodicity of 48 hours, on the contrary, should show negative correlation. A possible explanation for the difference between malariatherapy data and the data presented here could be that malariatherapy patients were not immune and therefore had fever more often. The question of periodicity in symptomatic malaria should be considered separately, and it's causes are thought to be well explained [20,21]: high temperature (fever) differentially affects the intra-erythrocytic stages of parasite development, and nearly stops development in some of these. This leads to "queuing" of the parasite population, and when the fever goes down, all parasites continue their development in a synchronized way. Fever can by definition not be operating in asymptomatic individuals, but at least in simian and avian malaria an effect of normal diurnal changes in body temperature on synchronization has been demonstrated, alongside with the observation that sometimes the parasite population is split into "two broods [..], coming to schizogony on alternate days" [22]. Two broods, synchronized within themselves, appearing in the peripheral blood with a 48 hour periodicity, yet with a 24 hour phase-shift, would appear in the data as having a 24 hour periodicity. This would be consistent with the finding that detection results one day apart are positively correlated. As the data does not contain smaller time intervals, however, any such periodicity cannot be distinguished from a simple gradual change in detectability.

Analysis of periodicity of clonal infections would ideally make use of long series of parasitological observations of untreated infections with short intervals, but few studies have collected such data, partly for ethical reasons. Exceptions include the malariatherapy datasets [23], the studies of Farnert *et al *[10,24] and Magesa *et al *[25] in Tanzania, and Bruce *et al *[19,26] from Papua New Guinea. Bruce *et al *aggregated data for paired observations with identical interval length and calculated the probability of detecting an infection at the second occasion, conditional on it being detected at the first occasion. This analysis suggests values of detectability similar to the estimates in the present study, with a six day periodicity. This periodicity was interpreted as signal of a 48-hour underlying cycle because the sampling interval was three days, which meant that six-day and two-day periodicity could not be distinguished. Similar analyses of the other available datasets would be of value.

### Measurement of detectability

A comparison of different approaches for estimating detectability found remarkably good agreement of the obtained numerical values. Of practical interest is the use of a direct method of estimating the detectability *q *from pairs of surveys by using the number of clones which were detected once (*n*_1_), or twice (*n*_2_):

$$q\approx \frac{2n_{2}}{n_{1}+2n_{2}}.$$

This approach was found to give very similar results as the more sophisticated methods, provided the underlying assumption is met: the number of successful detections must follow a binomial distribution. The statistical properties of the data, as assessed by a series of tests, suggest that if there is an interval of at least 7 days between consecutive surveys, it is safe to make these assumptions (see *H*_5_). Alternative methods [1,6-8] rely on the same two assumptions, yet are further incorporating models for the processes of acquisition and loss of infections. While those may themselves be of interest, the associated measurements of *q *may be affected by assumptions about acquisition and loss of infections. Direct estimation using M0 is therefore recommended as a simple and practical alternative, if only detectability is of interest, and if the interval between two surveys is short enough so acquisition and loss of infection clones can safely be excluded.

### Epidemiological significance of detectability

**Figure 3:**
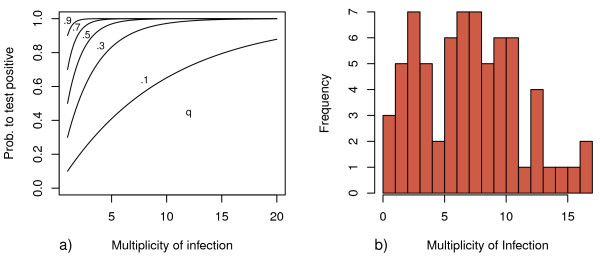
**The error in prevalence measurements becomes more important at low MOI**. a) Prevalence estimates are biased due to imperfect detection. Assuming that infecting clones within a particular host are independent from each other, the probability of missing all of them and therefore falsely classify an individual as negative, is highest for low multiplicity of infection. This graph shows - for different values of *q *- how the number of clonal infections in a host affects the estimates of prevalence. The probability of correctly recognizing a positive individual with n infections is calculated as 1 - (1 - *q*)*^n^*. It follows that the effect of detectability on prevalence estimates is highest at low multiplicity of infection and therefore low transmission, for example when being close to local elimination. However, low transmission intensity might prevent acquisition of immunity and therefore raise the value of detectability. It is therefore desirable to report estimates of *q *and multiplicity of infection together with prevalence estimates. b) The distribution of MOI. Contrary to common practice, observations from all four surveys are pooled for the calculation of MOI. This corresponds to the assumption that clones are present throughout all surveys if detected once. With the help of figure 3.a, the bias on prevalence estimates in this population, as introduced by imperfect detection, can be roughly estimated.

Prevalence and multiplicity of infection (MOI) are key epidemiological parameters, which characterize the malariological situation in a given area, and are routinely being reported. Quantities like these are ultimately important for rational planning of interventions. Both mentioned quantities are, however, affected by the value of detectability, which in comparison receives little attention. It seems plausible, that on average the "true" MOI should be the "observed" MOI divided by the detectability, which implies - given values of *q *around 0.5 - that true MOI's are roughly double of what is being reported. However, this ignores, that detectability itself might depend on MOI, and is merely an approximation. What about estimates of prevalence? It seems plausible that the extent to which measurements of prevalence are influenced by the value of *q *should vary with the multiplicity of infection, as the probability to miss every single one of *n *clones in a host (and obtain a false negative result) could be stated as (1 - *q*)*^n ^*(Figure 3).

This implies that the measurement error for prevalence, when neglecting detectability, should be highest at the lowest multiplicities of infection - a situation to be expected when approaching local elimination. It is therefore desirable to routinely report *q *together with other epidemiological measures, if possible.

In drug efficacy trials, the phenomenon of imperfect detection complicates the task of distinguishing new from breakthrough infections, and therefore must have an influence on drug efficacy estimates. In addition, residual drug levels may keep parasite densities at undetectable levels for some time, which is usually taken into account when designing drug efficacy trials. No satisfactory statistical methodology for analysis of such trials appears to exist, taking into account both imperfect detection and residual drug levels. It is suspected that many recrudescent infections, i.e. infection clones which survive treatment and are detected several days or weeks later, might in fact often be detected earlier if multiple testing took place. This is strongly supported by the findings of [27], who note that consecutive-day blood sampling changes the results of a drug efficacy trial compared to single-day blood sampling.

## Conclusions

The presented work demonstrates the importance of paying attention to the phenomenon of imperfect detection not only in the sense of assessing the sensitivity of diagnostic tests, but also looking at it as a property of infections or individuals. Various epidemiological measures, such as prevalence or MOI, are affected by imperfect detection. Failure to account for it may severely distort the outcome of measurements and may even lead to wrong conclusions. As an example, the decrease in prevalence with age, as it is frequently observed in malaria, might actually mean that detectability decreases with age, while prevalence remains constant or even increases. Indeed, some publications suggest that this might be the case [8]. In addition, it is likely that underestimation of prevalence may be substantial in situations where the multiplicity of infection is low. This is because the chance of missing every single clone in a host is highest when there are only few.

A simple method of estimating detectability from molecular data, using pairs of surveys, was presented. It is a modification of existing methods, which can deal with data on multiple infections within one host. The numerical estimates of detectability obtained using said formula appeared remarkably robust. Through comparison of the detectability estimates with estimates from different models, and through a series of statistical tests, the conditions under which the underlying assumptions of the method are fulfilled could be established. Its use is recommended when the time interval between the two surveys is one week or more, but discouraged on data with shorter time intervals, if possible. Both the method itself as well as the way of addressing it's applicability are not restricted to malaria, but may in a similar way be used for other infectious diseases where molecular data on individual clones is available.

The restrictions on applicability stem from the complicated dynamics of detectability on short time scales. These were investigated and it was found that treating individual detections as statistically independent is only an acceptable approximation for time intervals longer than one week. Contrary to expectation, however, no changes in detectability indicative of a 48 hour cycle were found, as is reported from malariatherapy data. This suggests that not the 48 hour erythrocytic cycle of *P*. *falciparum *is dominating detectability in vivo, but that other factors, such as e.g. the dynamics of the immune system, may be important. As the participants of the study must be considered partly immune, it is presumed that the within-host dynamics of infections differ between immune and non-immune individuals. This questions the use of malariatherapy data for fitting of within-host models for the immune host, and encourages further collection of relevant data as well as development of analysis methods in order to gain better insight into the within-host dynamics of *P*. *falciparum *in immune individuals.

## Competing interests

The authors declare that they have no competing interests.

## Authors' contributions

MTB performed the statistical analysis and drafted the manuscript. FV carried out the molecular genetic studies and helped drafting the manuscript. SO coordinated and carried out the data collection. MAP helped with the algebra needed for the statistical analysis. IF supervised the molecular genetic studies. TS carried out the study design, assisted in the statistical analysis, and helped drafting the manuscript. All authors read and approved the final manuscript.

## Supplementary Material

Additional file 1 **Appendix: estimating detectability using survey pairs**. This file contains a formal derivation of the mathematical expression used for direct estimation of detectability (M0). Click here for file
