# Supplementary material for: Detectability of Plasmodium falciparum clones
Source: Malar J. 2010 Aug 18;9:234. doi: 10.1186/1475-2875-9-234 (PMC2936403; doi:10.1186/1475-2875-9-234)
Supplement: Additional file 1 — Appendix: estimating detectability using survey pairs. This file contains a formal derivation of the mathematical expression used for direct estimation of detectability (M0). [file 1475-2875-9-234-S1.PDF]

Estimation of detectability from survey pairs

Michael Bretscher, Melissa A Penny, Tom Smith

A formula to estimate the detectability q from pairs of surveys has to take into account the fact, that double-negative pairs are never detected, and that therefore the data is biased. Such a formula was given by [1] for the diagnosis of onchocerciasis by microscopy. Here, the corresponding formula is derived for the context of genotyping data, where a human host can harbor multiple infections. The obtained algebraic expression in the case of two survey rounds will turn out to be identical to the one given in [1], despite the slightly different assumptions of the two approaches [1].

Assuming a binomial distribution of the number of successful detections, the probability that an infection produces a pair of observations with k positive results, p_k is

$$p_k = \frac{2}{k!(2-k)!} q^k (1-q)^{2-k}$$

A heuristic way of arriving at an estimator $\hat{q}$ of q is as follows: it is assumed that the actual data equal their expectations, i.e. $n_k = p_k n_{tot}$, of which n_1 and n_2 are known. Algebraically dividing n_2 by n_1,

$$\frac{n_2}{n_1} = \frac{q^2 n_{tot}}{2q(1-q)n_{tot}} = \frac{q}{2-2q},$$

and solving for q yields equation the desired result [2]:

$$\hat{q} = \frac{2n_2}{n_1 + 2n_2}. \tag{1}$$

[1] In the method of [1], n_k signifies the number of individuals testing positive k times in a study, and the prevalence is unknown. For the context of genotyping data, n_k denotes the total number of infections found in the study population which were detected k times, and the total number of infections in the study population is unknown.

[2] Through division by q the solution $q = 0$ is lost. It is not plausible for physical reasons, as one would then not observe any data.

Maximum likelihood estimation of q

A formal derivation of equation qhat makes use of a multinomial likelihood model and uses the probabilities of getting k successes conditional on the probability that an infection appears in the data[3], i.e. $\frac{p_k}{1-p_0}$. The likelihood of having n_1 single and n_2 double detections in $n_1 + n_2$ trials can then be written as follows:

$$\begin{aligned} L(q) &= \underbrace{\frac{(n_1+n_2)!}{n_1!n_2!}}_{=konst.} \left(\frac{p_1}{1-p_0}\right)^{n_1} \left(\frac{p_2}{1-p_0}\right)^{n_2} \\ &\propto \left(\frac{2q(1-q)}{1-(1-q)^2}\right)^{n_1} \left(\frac{q^2}{1-(1-q)^2}\right)^{n_2} \\ &\propto \left(\frac{2-2q}{2-q}\right)^{n_1} \left(\frac{q}{2-q}\right)^{n_2}. \end{aligned}$$

Omitting constant factors and taking the logarithm yields the log-likelihood function:

$$l(q) = n_1 \log(2-2q) - n_1 \log(2-q) + n_2 \log q - n_2 \log(2-q).$$

We obtain the score function $S(q)$ by taking the derivative of $l(q)$ with respect to q:

$$S(q) = \frac{d}{dq} l(q) = \frac{2n_2 - q(n_1 + 2n_2)}{q(1-q)(2-q)}$$

The maximum likelihood estimator $\hat{q}$ of q can then be determined by finding the root of the score function

$$\frac{2n_2 - \hat{q}(n_1 + 2n_2)}{\hat{q}(1-\hat{q})(2-\hat{q})} = 0.$$

This expression can only be zero, if the numerator is zero, and therefore it simplifies to

$$2n_2 - \hat{q}(n_1 + 2n_2) = 0.$$

Solving for $\hat{q}$ leads to equation (1),

$$\hat{q} = \frac{2n_2}{n_1 + 2n_2},$$

which confirms that it is a maximum likelihood estimator of q.

[3] A note on conditional probabilities: The probability that an event A occurs, given that an independent event B has already occurred, equals the probability that both events occur divided by the probability that B occurs, namely $P(A|B) = P(A,B)/P(B)$. This may at first not be obvious, but follows through simple rearrangement of the more familiar expression $P(A|B)P(B) = P(A,B)$.

Confidence interval

Construction of a confidence interval requires the Fisher information $I(q)$, which is the negative derivative of the score function, namely

$$I(q) = -\frac{d}{dq}S(q) = -\frac{d}{dq}\left(\frac{2n_2 - qn_1 - 2qn_2}{q(1-q)(2-q)}\right),$$

which leads to

$$I(q) = -\frac{n_1 + n_2}{(q-2)^2} + \frac{n_1}{(q-1)^2} + \frac{n_2}{q^2}. \tag{2}$$

The observed fisher information I_{obs} is $I(q)$ evaluated at $q = \hat{q}$, so

$$I_{obs} = -\frac{n_1 + n_2}{(\hat{q}-2)^2} + \frac{n_1}{(\hat{q}-1)^2} + \frac{n_2}{\hat{q}^2},$$

which simplifies to

$$I_{obs} = \frac{(n_1 + 2n_2)^4}{4n_1 n_2 (n_1 + n_2)}$$

This allows us to calculate the standard error of $\hat{q}$ as

$$SE(\hat{q}) = \frac{1}{\sqrt{I_{obs}}} = \frac{2\sqrt{n_1 n_2 (n_1 + n_2)}}{(n_1 + 2n_2)^2}.$$

A confidence 95% confidence interval for $\hat{q}$ can then be constructed using Wald's approximation:

$$[\hat{q} \pm 1.96 SE(\hat{q})]$$

References

[1] K. Mullen and A. Prost, "Decreased microfilarial load and its effect on the calculation of prevalence and the rate of false negatives in the detection of onchocerciasis," *Int J Epidemiol*, vol. 12, pp. 102–104, Mar. 1983. PMID: 6840949.
